# Effect of the hour-1 bundle on clinical outcomes in patients with sepsis and septic shock: A protocol for systematic review and meta-analysis

**DOI:** 10.1371/journal.pone.0318914

**Published:** 2025-02-06

**Authors:** Shukun Hong, Hongye Wang, Xiaoguang Fan, Jian Liu, Lujun Qiao

**Affiliations:** 1 Department of Intensive Care Unit, Shengli Oilfield Central Hospital, Dongying, China; 2 Clinical Research Center of Dongying Critical Care Medicine, Dongying, China; 3 Department of Obstetrics and Gynecology, Shengli Oilfield Central Hospital, Dongying, China; Azienda Ospedaliero Universitaria Careggi, ITALY

## Abstract

**Background:**

According to the 2018 bundle guidelines of the Surviving Sepsis Campaign, many emergency departments and intensive care units currently adopt the hour-1 bundle as a standard practice for sepsis management. However, recent studies on the hour-1 bundle for sepsis treatment have yielded inconsistent results, raising questions and challenges about its clinical efficacy.

**Aim:**

This study will conduct a systematic review and meta-analysis to compare the impact of the hour-1 bundle and non-hour-1 bundle on the clinical outcomes in patients with sepsis and septic shock.

**Methods:**

The protocol was prepared according to the guidelines of the Preferred Reporting Items for Systematic Reviews and Meta-analyses protocol (PRISMA-P) statement. The systematic review will be carried out in line with the statement of PRISMA. The following electronic databases will be searched: PubMed, EMBASE, Cochrane Central Register of Controlled Trials, and Web of Science. All clinical studies comparing the impact of the hour-1 bundle and non-hour-1 bundle on clinical outcomes in patients with sepsis and septic shock will be included. All stages of the literature search, study selection, data extraction, and quality assessment will be conducted independently by two reviewers. Any disagreements between the two reviewers will be resolved by discussion or arbitration by a third reviewer. The primary outcome will be short-term mortality, which involves in-hospital, 28-day, 30-day, and 90-day mortality corresponding to the definition used in each study. For quality assessment, the risk of bias specified by the Cochrane Collaboration and the methodological index for non-randomized studies will be used for randomized control trials (RCTs) and non-RCTs, respectively. Data synthesis will be performed via Review Manager 5.1.0.

**Expected results:**

This systematic review will integrate all relevant studies to quantitatively estimate the effect size and clarify the role of the hour-1 bundle in sepsis management, contributing new evidence-based guidance to the field.

**Systematic review registration:**

Protocol registration and reporting: PROSPERO CRD42024579314.

## Introduction

Sepsis is defined as life-threatening organ dysfunction caused by a dysregulated host response to infection [1, 2]. Sepsis and septic shock are major causes of death in emergency departments and intensive care units (ICUs) worldwide. Their complex pathophysiological processes and rapid progression lead to high mortality rates and significant healthcare resource consumption [3]. According to an analysis of the global burden of disease study, nearly 50 million new cases of sepsis occur annually, with approximately 10 million deaths [4]. Given the high incidence and mortality of sepsis, finding effective and timely treatment strategies is a hot and challenging area of global medical research.

For the past two decades, the Surviving Sepsis Campaign (SSC), launched by the European Society of Intensive Care Medicine (ESICM) and Society of Critical Care Medicine (SCCM), has been advocating that early identification and appropriate management in the initial hours after the development of sepsis improve outcomes [1, 2]. As recommended by the SSC guidelines, sepsis bundles have been widely recognized and applied in the international medical community [5, 6]. The core principle of bundles is to achieve timely recognition, rapid response, and multidisciplinary collaboration to implement standardized interventions to reduce sepsis mortality. Over time, the bundles have been updated based on new research evidence, transitioning from the original 6-hour bundle in 2004 [7, 8] to the 3-hour and 6-hour bundle in 2012 [9, 10], and then to the hour-1 bundle in 2018 [11, 12], reflecting a greater emphasis on early resuscitation and management in clinical practice. The hour-1 bundle elements include measuring lactate levels, obtaining blood cultures before antibiotics, using broad-spectrum antibiotics, rapid fluid resuscitation, and using vasopressors as needed. However, owing to the lack of strong evidence supporting certain elements of the bundles and challenges of implementation in different regions and healthcare systems, the hour-1 bundle has not been well accepted [13].

In recent years, several clinical studies have attempted to demonstrate the value of the hour-1 bundle in sepsis treatment [14–16]. Unfortunately, the results are inconsistent, raising concerns about its effectiveness and authority in sepsis management. To the best of our knowledge, there is currently no registered systematic review on the efficacy of hour-1 bundle therapy. In this context, we will conduct a systematic review and meta-analysis to compare the impact of the hour-1 bundle and non-hour-1 bundle on the clinical outcomes in patients with sepsis and septic shock, aiming to provide new evidence-based guidance for sepsis treatment.

## Methods

### Protocol registration and reporting

This study protocol was registered in the International Prospective Register of Systematic Reviews (PROSPERO) (CRD42024579314). The protocol was prepared according to the guidelines of the Preferred Reporting Items for Systematic Reviews and Meta-analyses protocol (PRISMA-P) statement [17]. The PRISMA-P checklist is revealed in S1 Table. The systematic review will be carried out in accordance with the statement of Preferred Reporting Items for Systematic Reviews and Meta-Analyses (PRISMA) [18]. Owing to the use of secondary data in this study, approval from the research ethics committee will not be needed.

### Eligibility criteria

#### Inclusion criteria

The eligibility criteria will be defined using Participants, Intervention, Comparison, Outcomes, Study design (PICOS) elements. All clinical studies comparing the impact of the hour-1 bundle and non-hour-1 bundle on clinical outcomes in patients with sepsis and septic shock will be included in the meta-analysis. Because the concept of the hour-1 bundle was proposed by the sepsis bundle guidelines published in April 2018, the publication time for the included studies will be limited to after April 1, 2018. There will be no language restrictions for publication.

#### Participants

We will include studies that compared the impact of adherence or non-adherence to the hour-1 bundle on clinical outcomes in patients with sepsis and septic shock.

#### Intervention

Patients in the “intervention” or “hour-1 bundle” group in the included studies received completed hour-1 bundle therapy. The hour-1 bundle of the SSC includes the following elements, all of which must be completed within 1 hour:

1. Measure lactate level. Remeasure if initial lactate is >2 mmol/L.
2. Obtain blood cultures prior to the administration of antibiotics.
3. Administer broad-spectrum antibiotics.
4. Begin rapid administration of 30 ml/kg crystalloid for hypotension or lactate≥4 mmol/L.
5. Apply vasopressors if patient is hypotensive during or after fluid resuscitation to maintain mean arterial pressure (MAP)≥65 mmHg.

#### Comparison

Patients in the “control” or “non-hour-1 bundle” group in the included studies received incomplete hour-1 bundle therapy or bundle therapy for more than 1 hour.

#### Outcomes

The primary outcome will be short-term mortality, which will involve in-hospital, 28-day, 30-day, and 90-day mortality corresponding to the definition used in each study. The secondary outcomes will include hospital stay, ICU stay, duration of mechanical ventilation, and ventilator-free days during hospitalization.

#### Study design

All scientific publications with the following characteristics will be included: randomized control trials (RCTs), cohort studies, case-control studies, quasi-experimental studies (before and after), and time series studies; compared hour-1 bundle with non-hour-1 bundle treatment for patients with sepsis or septic shock; and reported on at least one of the outcomes mentioned above.

#### Exclusion criteria

Reviews, case reports, letters, editorials, commentaries, and comparative studies that presented insufficient data will be excluded from the study. In cases of duplicates, the most recent or complete publication will be used. Publications that study individual elements in the bundle will also be excluded.

### Information sources and search strategy

All stages of the literature search, study selection, data extraction, and quality assessment will be conducted independently by two reviewers. Any disagreements between the two reviewers will be resolved by discussion or arbitration by a third reviewer. The literature search will employ several electronic databases to ensure that all available published literature will be included in our systematic review. The following electronic databases will be searched with no language or geographical restrictions: PubMed, EMBASE, Cochrane Central Register of Controlled Trials, and Web of Science. The search strategy used in PubMed is presented in Table 1 and will be changed appropriately depending on the rules of each database. All the references cited in the relevant articles will be screened to identify eligible studies. Since the concept of the hour-1 bundle was introduced in April 2018, the literature search will commence on April 1, 2018.

**Table 1:** Search for PubMed.

Search order	Search terms
1	Bundle [tiab]
2	"Patient Care Bundles" [Mesh]
3	"Early Goal Directed Therapy" [tiab]
4	"Early Goal-Directed Therapy" [Mesh]
5	OR/1-4
6	Sepsis [tiab]
7	"Sepsis" [Mesh]
8	Shock [tiab]
9	"Shock, Septic" [Mesh]
10	Septic [tiab]
11	OR/6-10
12	Mortality [tiab]
13	"Mortality"[Mesh]
14	Fatality [tiab]
15	"Death"[Mesh]
16	"Treatment Outcome"[Mesh]
17	"Length of Stay"[Mesh]
18	"Respiration, Artificial"[Mesh]
19	OR/12-18
20	5 AND 11 AND 19

### Study selection

**Fig 1:**
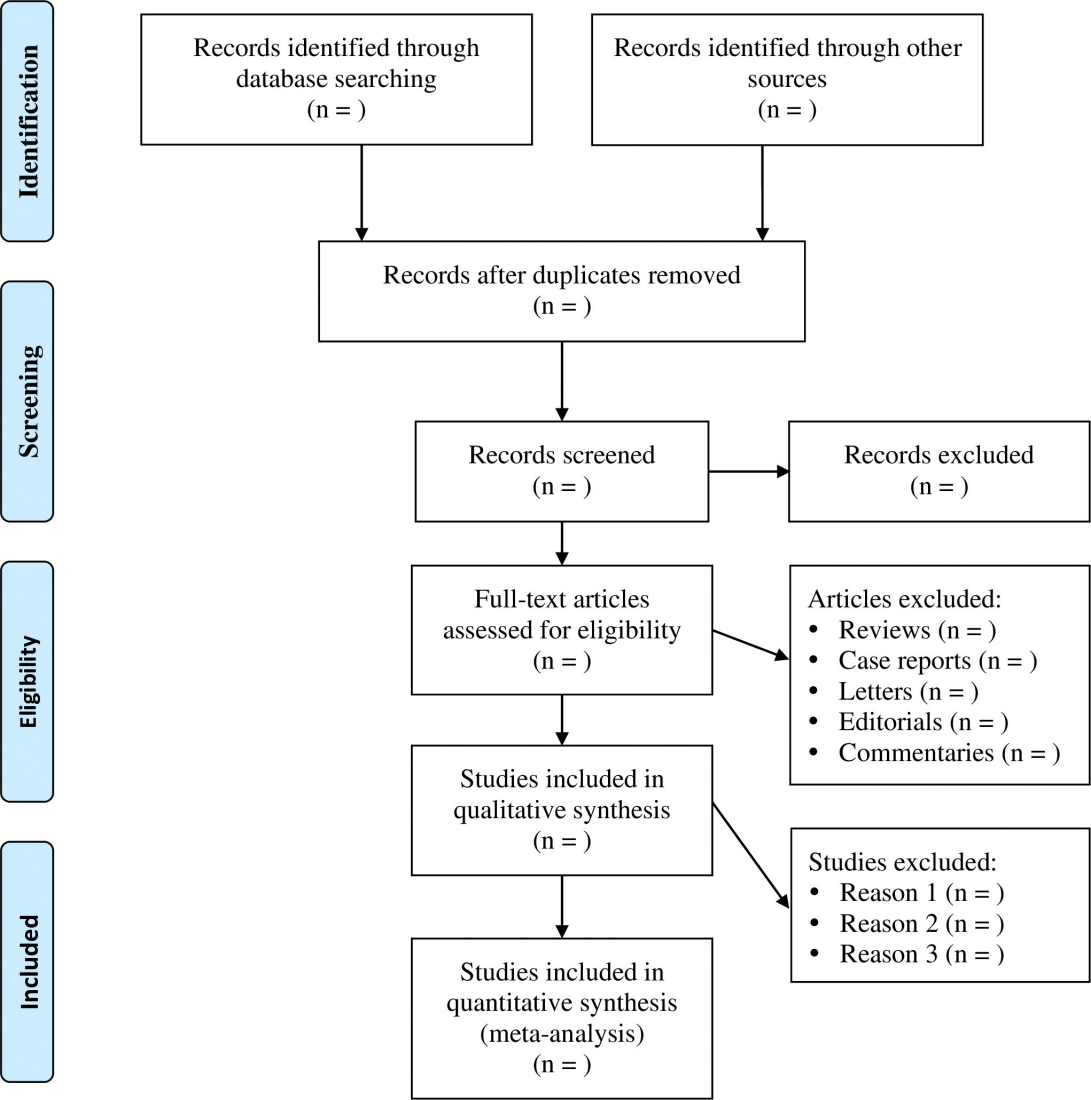
PRISMA flow diagram chart for selection of studies included in the systematic review.

All published clinical studies comparing the hour-1 bundle with non-hour-1 bundle for patients with sepsis or septic shock will be eligible. When two studies reported on a group of patients at the same institution with an overlapping time period, the article with the longest follow-up period will be included to avoid data duplication. Two independent reviewers will screen the titles and abstracts of the retrieved literature according to the inclusion criteria and then further evaluate the eligibility of citations by reading the full texts. The reasons for excluding citations will be documented during the review stages. The study selection process is shown in Fig 1.

### Data extraction and management

The following information will be extracted from each study via standardized data extraction forms: the first author’s last name; year of publication; study design; country; study interval; sample size; sex composition; mean age; severity of illness (e.g., APACHE and SOFA scores); study setting (i.e., emergency department, ICU or hospital ward); sepsis bundle therapy in each group; inclusion criteria; primary outcome; other study features; and data needed for quality assessment. The number of events and the total number of participants in each group will be used for extracting binary variables. The mean and standard deviation (SD) will be used to extract continuous variables. The metadata will be managed via Microsoft Excel (2010 version).

### Dealing with missing data

If the relevant data in the included literature are missing or unclear, the review team will try to contact the corresponding authors via email. If these efforts are unsuccessful, the data will be excluded from the meta-analysis, and this issue will be addressed in the discussion section.

### Risk of bias and quality assessment

The methodological quality of the RCTs will be assessed according to the criteria specified by the Cochrane Collaboration [19]. The assessed items of risk of bias involve random sequence generation (selection bias), allocation concealment (selection bias), blinding of participants and personnel (performance bias), blinding of the outcome assessment (detection bias), incomplete outcome data (attrition bias), selective reporting (reporting bias), and other bias. Each risk of bias will be classified as low risk, high risk, or unclear risk.

For non-RCTs, the methodological index for non-randomized studies (MINORS) will be used for quality assessment [20]. The MINORS scoring system includes the following items: a clearly stated aim, inclusion of consecutive patients, prospective collection of data, endpoints appropriate to the aim of the study, unbiased assessment of the study endpoint, follow-up period appropriate to the aim of the study, loss to follow up less than 5%, prospective calculation of the study size, an adequate control group, contemporary groups, baseline equivalence of groups, and adequate statistical analyses. Each item may be scored 0 (not reported), 1 (reported but inadequate), or 2 (reported and adequate).

### Certainty of evidence

The certainty of evidence for each outcome will be evaluated using the Grading of Recommendations Assessment, Development, and Evaluation (GRADE) approach with the GRADEpro software. Evaluation according to the GRADE approach will be based on the risk of bias, inconsistency, indirect evidence, inaccuracies, and publication bias. Therefore, the certainty rate of the evidence will be categorized as high, moderate, low, or very low [21].

### Data synthesis

The outcomes will be pooled as an estimate of the overall effect for the meta-analysis conducted via Review Manage, version 5.1.0 (The Cochrane Collaboration, 2011). As we previously reported [22, 23], for dichotomous variables, the pooled risk ratio (RR) with a corresponding 95% confidence interval (CI) will be aggregated in Mantel–Haenszel method, and the mean difference (MD) with a corresponding 95% CI will be calculated in inverse variance method for continuous variables. When continuous data are reported as a median and range, we will calculate the mean and SD using the method of Hozo et al. [24]. Clinical heterogeneity will be discussed when appropriate. Statistical heterogeneity will be assessed by Cochran’s *Q* test and *I*^*2*^ statistic, with *p*<0.1 or *I*^*2*^ > 50% considered significant. An *I*^*2*^ value of 0% indicates no observed statistical heterogeneity. If the statistical heterogeneity is not significant, then the fixed-effect model will be used; otherwise, the random effects model will be applied. Subgroup analysis and meta-regression will be implemented to help identify potential sources of heterogeneity if there is significant heterogeneity across studies. For subgroup analyses, we will conduct them based on the characteristics of the included studies, including single-center versus multicenter studies, prospective versus retrospective studies, sepsis without shock versus septic shock, and study settings such as the emergency department and ICU. Sensitivity analysis will be carried out, if applicable, by excluding studies to remove heterogeneity when it is statistically significant. For example, in such analyses, we will stratify the meta-analysis by study types—experimental versus observational studies—to assess any changes in the results. A forest plot will be constructed to graphically assess the statistical heterogeneity by displaying effect estimates and 95% CIs for both individual studies and meta-analyses. The *p*-value threshold for statistical significance will be set at 0.05 for effect sizes. Publication bias will be explored by Begg’s funnel plot and Egger’s regression test, with *p*<0.05 considered significant (STATA 12.0).

## Discussion

The SSC guidelines advocate that the management of sepsis primarily relies on early identification and timely appropriate treatment [1, 2]. The expected effect of the bundle recommended by the guidelines is to stabilize hemodynamics, improve microcirculatory perfusion, and stabilize the internal environment. Most medical institutions have standardized bundle measures and achieved better clinical practice outcomes. However, despite this, the mortality rate of sepsis and septic shock remains relatively high [4]. To clearly indicate that resuscitation and management should begin immediately upon sepsis diagnosis, the 3-hour and 6-hour bundles were combined into a single hour-1 bundle in the 2018 SSC bundle guidelines [11, 12]. Since the release of the new bundle, its clinical utility has been challenged and questioned. Based on the bundle guidelines, the evidence level for each element ranges from low to moderate. There is a lack of data for investigating the hour-1 bundle as a whole measure. After all, the overall clinical effect of the hour-1 bundle is not a simple superposition of the effects of each element. Accordingly, many researchers have attempted to address this issue by conducting clinical studies.

A multicenter retrospective study on septic shock patients from Korea revealed that there was no significant difference in in-hospital mortality between the group that achieved the hour-1 bundle and the group that did not achieve the hour-1 bundle (<1 vs. >1 h) [14]. Nevertheless, this study also revealed that 3- and 6-h bundle achievements were related to significantly lower odds ratios of in-hospital mortality compared to the group that did not achieve the bundle (<3 vs. >3 h, <6 vs. >6 h). Similar reports can be found in a multicenter RCT conducted jointly by France and Spain [15]. The trial recruited suspected sepsis patients from emergency departments and concluded that the implementation of the hour-1 bundle was not associated with a significant difference in in-hospital mortality. In contrast, multicenter retrospective data from New York State showed that completion of the hour-1 bundle was associated with reduced risk-adjusted in-hospital mortality compared with not completing the hour-1 bundle among patients with pediatric sepsis and septic shock [25]. This result is consistent with a multicenter retrospective study conducted in Japan [16]. Thus it can be seen that the effectiveness of hour-1 bundle therapy in sepsis and septic shock treatment is controversial. The current evidence in the literature cannot provide reliable references for clinical physicians. Under such an environment, we will conduct the first systematic review and meta-analysis on this topic by integrating all relevant studies to increase the statistical power, quantitatively estimate the average level of research effects, and evaluate the inconsistency of research results. We hope our research can contribute new evidence-based insights to this field.

Our systematic review was initially designed to include only RCTs for meta-analysis. However, we recognize that RCTs on this topic may be scarce. After all, most hospitals currently follow SSC guidelines for sepsis management. Therefore, we will include both RCTs and non-RCTs to increase the sample size. Meanwhile, in the selection process of the included studies, we will strictly control to minimize bias.

In anticipation of conducting this meta-analysis, we recognize several potential limitations that may arise if the certainty of evidence is found to be low or very low. Such limitations could include the quality and consistency of data across studies, the potential for publication bias, and the presence of significant heterogeneity. We acknowledge that heterogeneity across studies may affect the interpretation of our results, and we plan to conduct subgroup analyses and sensitivity analyses to explore its sources. Factors contributing to heterogeneity may include differences in study design, patient populations, and the implementation of interventions. To mitigate these challenges, we will adhere to rigorous methodological standards, including the use of the GRADE approach to assess the certainty of evidence and the application of robust statistical methods to handle heterogeneity. In addition, the outcomes of interest reported in this systematic review may be limited. Some outcomes that may be of interest to clinical physicians might not be included in the present review, mainly because of the original descriptions of the included studies. If more results are found to be reportable during the formal review, we will make appropriate modifications to the protocol.

In summary, this systematic review will integrate all relevant studies to quantitatively estimate the effect size and clarify the role of the hour-1 bundle in sepsis management, contributing new evidence-based guidance to the field.

## Supporting information

S1 Table PRISMA-P 2015 checklist. (DOC)
